# The role of *Leptospira* spp. in horses affected with recurrent uveitis in the UK

**DOI:** 10.1111/evj.12683

**Published:** 2017-04-24

**Authors:** F. Malalana, R. J. Blundell, G. L. Pinchbeck, C. M. Mcgowan

**Affiliations:** ^1^ School of Veterinary Science, University of Liverpool Neston UK; ^2^ Institute of Infection and Global Health School of Veterinary Science, University of Liverpool Neston UK; ^3^ Institute of Ageing and Chronic Disease School of Veterinary Science, University of Liverpool Neston UK

**Keywords:** horse, ERU, seroprevalence, aqueous humour, antibody titre

## Abstract

**Background:**

Equine recurrent uveitis (ERU) is a common cause of ocular pain and blindness in horses. *Leptospira* spp. have been commonly implicated in the pathophysiology of ERU in mainland Europe and the USA. No recent studies have been carried out in the UK, but *Leptospira* is reported not to be a major factor in the aetiology of ERU in the UK.

**Objectives:**

To establish the prevalence of *Leptospira*‐associated ERU in the UK and to identify the serovars involved in these cases; to compare serum vs. aqueous humour antibody levels in cases and controls in order to confirm the diagnosis of *Leptospira*‐associated ERU, and to assess the usefulness of serology alone as a confirmatory test for *Leptospira*‐associated ERU in the UK.

**Study design:**

Case–control study.

**Methods:**

Eyes enucleated for clinical reasons in ERU‐affected horses were collected. Blood and aqueous humour were obtained to determine antibody levels against a variety of *Leptospira* serovars and C‐values (aqueous humour value/serum value) were calculated. In addition, eyes, blood and aqueous humour were obtained from control cases for comparison. Histopathology was performed in all eyes to confirm uveitis in each case. Differences in seroprevalences between ERU and control cases and between *Leptospira*‐ and non‐*Leptospira*‐associated ERU cases were calculated.

**Results:**

A total of 30 ERU and 43 control eyes were analysed. Of the ERU eyes, only two had a C‐value of >4 (prevalence of *Leptospira*‐associated uveitis: 6.7%). Serovars *hardjo* and *javanica* were detected. There was no difference in seroprevalence between horses with uveitis and control cases (65.5% and 41.9%, respectively; P = 0.11) or between *Leptospira*‐ and non‐*Leptospira*‐associated uveitis cases (100% and 63.0%, respectively; P = 0.52).

**Main limitations:**

The study was limited by low case numbers. Eyes were presented at different stages of disease. The only test used to detect *Leptospira* was the microscopic agglutination test.

**Conclusions:**

*Leptospira‐*associated ERU is uncommon in the UK. Serology alone may not help to definitively diagnose *Leptospira*‐associated uveitis in this country.

## Introduction

Equine recurrent uveitis (ERU) is a disease characterised by repeated episodes of intraocular inflammation and is a common cause of ocular pain in horses 1. ERU is considered an autoimmune disease 2, but the initiating events of the disease remain unclear. *Leptospira* spp. have been commonly implicated in the pathogenesis of ERU, either by causing persistent intraocular infection 3, 4, 5, 6, 7, 8, 9, 10 or by triggering an immune response through antigenic molecular mimicry in a number of ocular structures 11, 12, 13, 14. A number of *Leptospira* serovars (mainly *grippotyphosa*,* pomona* and *bratislava*) have been implicated in the pathophysiology of ERU in Europe and the USA 3, 6, 8, 12, 15, 16, 17, 18. It is commonly believed that *Leptospira* infection is not a major factor in the aetiology of ERU in horses in the UK 19, 20, 21, 22.

Reported prevalences of ERU vary enormously among different geographic locations. In the USA, ERU is estimated to affect 2–25% of horses 16, 23. In mainland Europe, reported prevalences have varied from 7–10% to as high as 70% 24, 25, 26. The prevalence of ERU in the UK has not been accurately determined, but it is believed to be much lower 22, 27, 28 and a recent survey suggests a prevalence of 0.3% 29. The reasons for this much lower prevalence in the UK may reflect genetic differences in the horse population. A relationship between horse breed and risk for the development of uveitis has been established in Appaloosas and German Warmbloods 16, 30, 31. Another reason for this lower prevalence may be differences in the *Leptospira* serovars present in the UK and possibly lower environmental levels of the *pomona* and *grippotyphosa* serovars in the UK 22.

Exposure to *Leptospira* spp. is common in horses 32, but serum antibody titres do not correlate well with ocular signs. No recent studies have looked specifically at the presence of *Leptospira* in uveitis cases in the UK, and previous studies have relied on serum antibody levels alone 20. Aqueous humour combined with serum titres are a better indicator of *Leptospira*‐associated uveitis 12, 32, 33.

The aims of this study were to: 1) establish the prevalence of *Leptospira*‐associated ERU in animals in the UK; 2) recognise the serovars most commonly involved in *Leptospira*‐associated ERU cases in the UK; and 3) compare the value of serum vs. aqueous humour antibody levels against *Leptospira* spp. in order to confirm the diagnosis of *Leptospira*‐associated ERU and to assess the usefulness of serology alone as a confirmatory test for *Leptospira*‐associated ERU in the UK.

## Materials and methods

Eyes enucleated for clinical reasons in horses affected by ERU were collected over the period from April 2013 to June 2016. Eyes were considered as suffering from ERU if they showed any of the three clinical forms described 1: classic (two or more episodes of intraocular inflammation followed by periods of quiescence); insidious (persistent, low‐grade intraocular inflammation), and posterior (affecting mainly the vitreous, choroid and retina). Single‐episode uveitis or uveitis cases of traumatic origin were not included in this study. The study eyes were sourced from horses located within northwest England and northern Wales, and included materials sourced from the authors’ institution and referring veterinary practices. Blood was obtained from the jugular vein to determine serum antibody levels against a variety of *Leptospira* serovars using the microscopic agglutination test (MAT).^1^ The serovars analysed included *canicola*,* copenhageni*,* ballum*,* icterohaemorrhagiae*,* pomona*,* mozdok*,* tarassovi*,* grippotyphosa*,* australis*,* bratislava*,* autumnalis*,* hebdomadis*,* mini*,* sejroe*,* javanica*,* bataviae*,* zanoni* and *hardjo*. Samples were considered positive when agglutination was obtained at a dilution of 1:100. Samples of aqueous humour were also collected from each affected eye and analysed for *Leptospira* antibody levels to the same serovars, and the Goldmann–Witmer coefficient or C‐value (aqueous humour antibody titre value/serum antibody titre value) was determined for each eye. A C‐value of >4 suggests specific intraocular production of antibodies rather than leakage through a damaged blood–ocular barrier and supports a diagnosis of *Leptospira*‐associated ERU 33. In order to confirm that ERU pathology was present, histopathological examination of all but one of the affected eyes was carried out. The eye in which histopathologic examination was not performed belonged to a horse that presented with signs of ERU, in which aqueocentesis was performed as part of the diagnostic work‐up, but the eye was not enucleated.

In addition, eyes, blood and aqueous humour samples were obtained in an identical manner from control horses for comparison. Controls included horses that were subjected to euthanasia for reasons unrelated to this study within the same region during the same period. A full ocular examination was carried out in a randomly allocated eye prior to euthanasia to exclude any horses with previous or ongoing signs of ocular disease, and all eyes were submitted to histopathology to rule out any ocular pathology.

For histopathological examination, eyes were prepared as follows. Subsequent to enucleation, all extraocular tissues were removed. A sample of aqueous humour was obtained, after which 1 mL of 10% formalin was injected into the anterior segment with a 25 gauge needle inserted at the level of the limbus. Another 1.5 mL of 10% formalin was injected into the posterior segment using a 25 gauge needle inserted at the level of the optic nerve. The whole globe was then placed in 10% formalin and fixed. Following fixation, eyes were sampled in two or three blocks (depending on size) and stained with haematoxylin and eosin.

Histological criteria for uveitis were the presence of lymphoplasmacytic infiltration within the ciliary body and/or choroid, with or without lymphoid follicle formation, fibrovascular membranes, cataract formation (globules of Morgagni), retinal detachment and/or optic nerve inflammation 33. The histological criterion for inclusion within the control group was the absence of any ocular disease.

The normality of continuous data was analysed with a Shapiro–Wilk test. Both populations were analysed for differences in age (Student's *t* test), and gender and eye investigated (Chi‐square test). Differences in seroprevalence (positive vs. negative) between ERU‐affected horses and controls, and between cases associated and not associated with *Leptospira* were evaluated using Chi‐square or Fisher's exact tests (for variables in which n≤5). Differences in antibody titres between ERU‐affected horses and controls, and between ERU cases associated and not associated with *Leptospira* were assessed with the Mann–Whitney test. A P<0.05 was considered to indicate statistical significance. IBM SPSS Statistics for Windows Version 21.0 was used for the statistical analysis.^2^

## Results

Thirty eyes from 29 horses affected with ERU were analysed (one horse with bilateral ERU was subjected to euthanasia and both eyes were analysed) (Supplementary Item 1). In addition, 43 control eyes were obtained for comparison (Supplementary Item 2). The mean ± s.d. age of the horses was 11.4 ± 5.0 years in horses in the control group 11.2 ± 4.6 years in those in the ERU‐affected group. There were 19 mares and 24 geldings in the control group, and 11 mares, 18 geldings and one stallion in the ERU group. There were 22 right and 21 left eyes in the control group, and 15 right and 15 left eyes in the ERU group. Both groups were comparable in terms of age (P = 0.9), sex (P = 0.4) and eye affected (right vs. left, P = 0.9).

**Figure 1:**
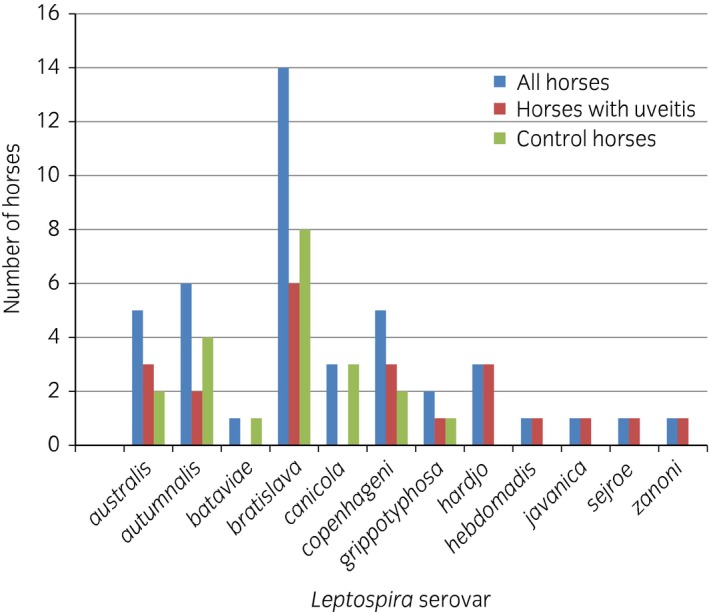
Results of serology to a variety of *Leptospira* serovars. No horses in the present study had any detectable antibody levels in serum to serovars *ballum*,* icterohaemorrhagiae*,* mini*,* mozdok or pomona*.

Of the 72 horses analysed, 37 were seropositive to one or more *Leptospira* serovars (51.4%, 95% confidence interval [CI] 40.1–62.6%). The *Leptospira* serovars detected most commonly were *bratislava* (14 horses), *autumnalis* (six horses), *copenhageni* and *australis* (five horses each) (Fig 1).

**Figure 2:**
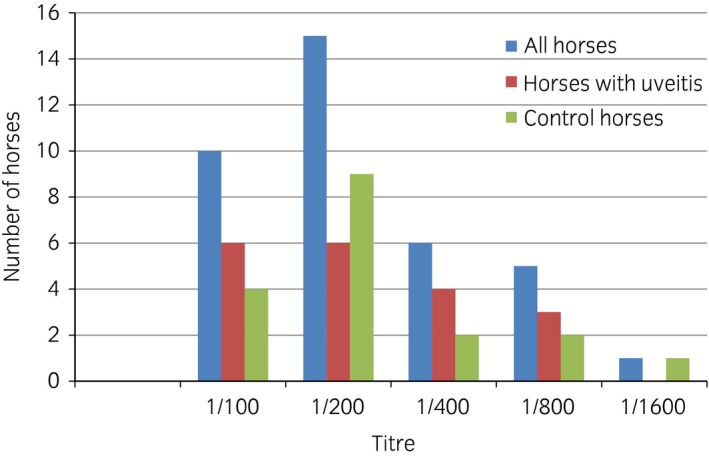
*Leptospira* spp. serum titres.

Nineteen of the 29 (65.5%, 95% CI 47.3–80.1%) horses with ERU‐affected eyes were seropositive to *Leptospira* spp. compared with 18 of the 43 (41.9%, 95% CI 28.4–56.7%) horses without uveitis. No differences between horses affected by ERU and controls emerged in overall seroprevalence to *Leptospira* spp. (positive vs. negative, P = 0.1) or antibody titre against *Leptospira* spp. (P = 0.1) (Fig 2).

Only six of the aqueous humour samples had detectable antibodies against *Leptospira* spp. All of these six eyes belonged to ERU‐affected animals. A positive antibody titre in the aqueous humour was significantly associated with a diagnosis of ERU (P = 0.002). C‐values were calculated for these six eyes. Only two of the horses had a C‐value of >4, indicating a prevalence of *Leptospira*‐associated ERU of only 6.7% (95% CI 1.8–21.3%). The serovars involved in the two eyes with C‐values of >4 were *hardjo* and *javanica*. Amongst the ERU cases there was no statistically significant difference in seroprevalence (P = 0.52) between *Leptospira*‐associated cases (those with a C‐value of >4) and those not associated with *Leptospira*, which indicates that blood antibody titres alone may not be helpful in the diagnosis of *Leptospira*‐associated ERU.

Histopathological examination did not detect any abnormalities in any of the control eyes. All of the affected eyes had findings consistent with ERU as previously described 34. The most common findings were lymphocytic inflammation of the ciliary body and choroid, lymphoid follicular formation, uveal haemorrhage and retinal atrophy and/or detachment.

## Discussion

This is the first study to look at the presence of aqueous humour as well as serum antibody titres to *Leptospira* spp. in ERU cases in the UK. For the purposes of analysis and comparison, the present authors chose agglutination at a dilution of 1:100 as indicating a positive result on MAT as this appears to be the most common value used in the equine literature, and was also the value used in previous studies carried out in the UK 19, 20. With this definition, prevalences of seropositivity in the animals included in this study were 41.9% in the control group and 65.5% in ERU‐affected horses, which are similar to the levels reported by Hathaway *et al*. (34.6% and 76.5%, respectively) 19, but much higher than those reported by Matthews *et al*. (9.4% and 11.1%, respectively) 20. Like Matthews *et al*. 20, the present group did not find any statistically significant difference in seroreactivity between ERU‐affected and control horses, indicating that measuring serum antibody titres in horses with ERU may not be of any benefit in the UK. This result contrasts with those of studies conducted in the USA that not only found differences in antibody titres between ERU‐affected and non‐affected horses, but also reported differences in the severity of disease, with seropositive ERU‐affected horses 4.4 times more likely to lose their vision than seronegative horses 15, 16. The estimated prevalence of *Leptospira*‐associated ERU of 6.7% in the present study is much lower than those reported in mainland Europe and the USA. Studies in Germany have shown that significant titres of intraocular antibodies against *Leptospira* spp. can be found in up to 94% of ERU cases 8, 18, 35. In the USA, results vary according to geographic location and indicate that *Leptospira* spp. are likely to play important roles in some areas 4, 9, 10, but not in others 12. Some studies also show differences depending on the *Leptospira* serovar involved, with serum antibody titres against the serovars *pomona*,* grippotyphosa* and *hardjo* being significantly associated with the presence of ERU in comparison with other serovars 4, 15. The serovars present in ERU eyes in the current study contrasted with findings elsewhere. In North America, the serovar most commonly associated with ERU is *pomona*, whereas *grippotyphosa* is most commonly implicated in cases in Europe 3, 6, 8, 15, 16, 17, 18, 23, 32. No antibodies to the serovar *pomona* were detected in the present study, and only two horses were seropositive to *grippotyphosa* (one control and one ERU case). In the two cases with suspected *Leptospira*‐associated ERU in the present study, the serovars involved were *hardjo* and *javanica*. Neither of these two horses had travelled outside the UK during their lifetimes. In the study by Matthews *et al*., all seropositive cases of ERU in the UK shared the serovar *sejroe* 20, whereas *sejroe* was detected in the serum of only one of the ERU cases and none of the controls in the present study.

In addition to variations in the serovars present in each geographic location, genetic diversity may also play a part in the differences in prevalences of ERU between the USA, continental Europe and the UK. Genetic analysis has identified a number of markers in Appaloosa horses and German Warmbloods that increase their susceptibility to the development of ERU 30, 31, 36. No genetic studies with regard to ERU have been carried out in the UK horse population.

In the present study, a positive serum antibody titre was not associated with the presence of a C‐value of >4 in ERU‐affected horses, which suggests that serology alone may not help to discriminate between *Leptospira*‐associated and non‐*Leptospira*‐associated ERU in this population. This is important because treatment options can vary according to the initiating cause of the intraocular inflammation. Pars plana vitrectomy (PPV), a procedure in which the ocular media, cells and inflammatory mediators are removed from the posterior segment, has been recommended for *Leptospira*‐associated cases and has achieved good success rates 37, 38. Case selection is essential because of the potential for serious complications following PPV; the present study suggests that only a small percentage of animals in the UK would benefit from this surgical technique and aqueocentesis should be an essential part of the diagnostic work‐up if PPV is under consideration.

This study has some limitations. Only 30 ERU‐affected eyes and 43 control eyes were analysed during the course of the study. Ideally, a larger sample of horses would have been analysed to detect differences in seroprevalences between ERU‐affected and control cases, and therefore the present results should be interpreted with caution and should be considered indicative rather than confirmatory. Horses in this study presented in different stages of disease; some eyes were enucleated relatively early in the course of disease as a result of marked disease severity and lack of response to treatment, whereas others were presented for enucleation in end‐stage disease subsequent to more insidious, low‐grade inflammation that had developed over a number of years. However, following exposure to *Leptospira* spp., antibodies can be detected for up to 7 years after infection 4, 39 and it is unlikely that any of the horses in the study would have had clinical disease for longer than this period. By contrast with other studies in which other techniques to identify the presence of *Leptospira* were used, in the present study only MAT was used for antibody detection. In a study by Brandes *et al*. 8, evidence of *Leptospira* involvement was detected by PCR in the vitreous of all eyes subjected to vitrectomy. However, these results were similar to those obtained by MAT (94%) and both were higher than those obtained using other techniques, such as culture (75%) or electron microscopy (24%) 8. Hence, it would appear that MAT is a sufficiently sensitive diagnostic modality for the detection of *Leptospira*‐associated ERU. Another potential problem with MAT is that antibodies in serum may cross‐react, which makes the determination of the exact serovars involved unsuccessful 39. This, however, seems to be a problem only during the acute infection phase of the disease and thus it is unlikely that this cross‐reactivity may have affected the results in the current study.

In summary, although *Leptospira* may play a role in some cases of ERU in horses in the UK, based on the results of the present study, its prevalence in ERU eyes appears to be low. In addition, the serovars involved in the cases in this study differed from those affecting horses in other geographic locations. This study also suggests that serology alone may not help differentiate between *Leptospira*‐associated and non‐*Leptospira*‐associated ERU cases, and aqueocentesis is probably necessary to confirm the diagnosis of *Leptospira*‐associated ERU in the UK.

## Authors’ declaration of interests

No competing interests have been declared.

## Ethical animal research

The study was approved by the University of Liverpool Veterinary Ethics Committee (ref. VREC150). Owners of the horses involved in the study provided written consent.

## Sources of funding

This study was partly funded by PetPlan Charitable Trust.

## Authorship

F. Malalana contributed to the study design and to data collection, analysis and interpretation. R.J. Blundell contributed to the data analysis and interpretation. G.L. Pinchbeck and C.M. McGowan contributed to the study design and to data analysis and interpretation. All authors contributed to the preparation of the paper and approved the final manuscript for publication.

## Supporting information


**Supplementary Item 1:** Horse signalment, serum and aqueous humour antibody titres (using a microscopic agglutination test) and calculated C‐values (where applicable) in uveitis‐affected eyes (n = 30).

**Supplementary Item 2:** Horse signalment, serum and aqueous humour antibody titres (using a microscopic agglutination test) and calculated C‐values (where applicable) in control eyes (n = 43).

## Manufacturers' addresses

1 Animal and Plant Health Agency, Laboratory Services Weybridge, Addlestone, UK.

2 IBM Corporation, Armonk, New York, USA.
