# Supplementary material for: The role of Leptospira spp. in horses affected with recurrent uveitis in the UK
Source: Equine Vet J. 2017 Apr 24;49(6):706–9. doi: 10.1111/evj.12683 (PMC5655720; doi:10.1111/evj.12683)
Supplement: Supplementary file 1 — Supplementary Item 1: Horse signalment, serum and aqueous humour antibody titres (using a microscopic agglutination test) and calculated C‐values (where applicable) in uveitis‐affected eyes (n = 30). [file EVJ-49-706-s001.pdf]

Equine Veterinary Journal

Supplementary Item 1: Horse signalment, serum and aqueous humour antibody titres (using a microscopic agglutination test) and calculated C-values (where applicable) from uveitis affected eyes (n = 30).

y = years; m = months; x = cross, L = left, R = right, - = no antibodies detected to serovars no antibodies detected to serovars *canicola, copenhageni, ballum, icterohaemorrhagiae, pomona, mozdok, tarassovi, grippotyphosa, australis, bratislava, autumnalis, hebdomadis, mini, sejroe, javanica, bataviae, zanoni* and *hardjo*. Samples were considered positive when agglutination was obtained at dilution of 1:100.

Eye	Age	Breed	Eye	Serum titre	Aqueous titre	C value	Serovar(s)involved	Type of ERU case
1	11y	Welsh x	L	-	-			insidious
2	8y	Cob x	L	1/400	1/1600	4	*hardjo*	classic
3	11y	Welsh	L	-	-			classic
4	11y	Welsh	R	-	-			classic
5	14y	WB	R	1/100	1/100	1	*grippotyphosa*	insidious
6	14y		L	1/400	-		*bratislava*	classic
7	12y	Arab	R	-	-			classic
8	9m	Friesian	R	-	-			classic
9	11y	TB	R	-	-			classic
10	11y	WB	R	1/400	-		*bratislava*	classic
11	16y	Welsh x	L	1/400	-		*copenhageni*	classic
12	17y		L	1/100	-		*bratislava / autumnalis*	classic
13	4y	Cob	R	1/800	1/1600	2	*australis*	classic

14	15y	TB	L	1/100	-		*hardjo / sejroe*	classic
15	6y	Cob	R	-	-			classic
16	16y	TB x	R	1/200	-		*bratislava*	insidious
17	13	TB	R	1/200	1/12800	64	*javanica*	classic
18	11y	TB	R	1/100	-		*copenhageni*	classic
19	13y	TB x	L	1/100	-		*zanoni*	classic
20	18y	TB x	L	-	-			classic
21	11y	Cob	L	1/800	-		*copenhageni / australis*	classic
22	7y	KWPN	R	1/200	-			posterior
23	5y	Lusitano	R	1/200	1/800	4	*hebdomadis*	classic
24	14y	TB x	L	-	-			classic
25	16y	Fell Pony	R	1/200	-		*autumnalis*	insidious
26	12y	Welsh x	R	1/200	-		*bratislava*	classic
27	12y	Cob	L	-	-			classic
28	18y	Irish Sports	L	1/800	-		*australis / bratislava*	classic
29	5y	Welsh D	L	1/100	1/1600	16	*hardjo*	classic
30	2y	Cob	L	-	-			classic
