# Supplementary material for: The role of Leptospira spp. in horses affected with recurrent uveitis in the UK
Source: Equine Vet J. 2017 Apr 24;49(6):706–9. doi: 10.1111/evj.12683 (PMC5655720; doi:10.1111/evj.12683)
Supplement: Supplementary file 2 — Supplementary Item 2: Horse signalment, serum and aqueous humour antibody titres (using a microscopic agglutination test) and calculated C‐values (where applicable) in control eyes (n = 43). [file EVJ-49-706-s002.pdf]

Equine Veterinary Journal

Supplementary Item 2: Horse signalment, serum and aqueous humour antibody titres (using a microscopic agglutination test) and calculated C-values (where applicable) from control eyes (n = 43).

y = years; x = cross, L = left, R = right, - = no antibodies detected to serovars no antibodies detected to serovars *canicola, copenhageni, ballum, icterohaemorrhagiae, pomona, mozdok, tarassovi, grippotyphosa, australis, bratislava, autumnalis, hebdomadis, mini, sejroe, javanica, bataviae, zanoni* and *hardjo*. Samples were considered positive when agglutination was obtained at dilution of 1:100.

Eye	Age	Breed	Eye	Serum titre	Aqueous titre	C value	Serovar(s) involved
1		TB x	R	1/100	-		*bratislava / autumnalis*
2	1y		L	1/100	-		*bratislava*
3	9y	Welsh x	L	-	-		
4	14y	TB x	R	1/100	-		*copenhageni*
5	11y	Welsh x	L	-	-		
6	14y	TB x	R	1/400	-		*canicola*
7	5y	Pony	L	1/200	-		*bataviae*
8	15y	TB x	R	1/200	-		*autumnalis*
9	14y	WB x	R	-	-		
10	17y	Welsh x	R	1/200	-		*bratislava*
11	17y	Irish Dr.	R	1/200	-		*autumnalis*
12	15y	WB	R	1/800	-		*bratislava / autumnalis*
13	5y	Andalu.	L	1/400	-		*bratislava*
14	4y	Irish Sp.	R	-	-		
15	11y	Irish Sp.	R	-	-		
16	6y	Appalo.	R	-	-		
17	5y	Cob	R	-	-		
18	10y	TBx	R	-	-		
19	12y	TB	L	-	-		
20	11y	Cob	R	-	-		
21	15y	Welsh x	L	-	-		
22	10y	TB x	R	1/100	-		*copenhageni*
23	15y	Cob	R	-	-		

24	12y	Cob	L	-	-		
25	14y	TB	L	-	-		
26	11y	WB	R	-	-		
27	12y	Welsh x	L	-	-		
28	15y	Welsh x	R	-	-		
29	16y	TB	L	-	-		
30	11y	TB x	L	-	-		
31	15y	Cob	L	1/800	-		*australis*
32	25y	Cob	L	-	-		
33	16y	Conn x	L	-	-		
34	18y	Camarg	L	1/200	-		*grippotyphosa*
35	14y	Friesian	L	1/200	-		*canicola*
36	7y	Irish Sports	R	-			
37	4y	WB	L	-	-		
38	16y	Pony	R	1/200	-		*canicola*
39	6y	Irish Sports	L	1/200	-		*australis / bratislava*
40	10y	Welsh D	L	1/1600	-		*bratislava*
41	3y	TB	R	-	-		
42	13y	Irish Sports	R	1/200	-		*bratislava*
43	4y	Welsh x	L	-	-		
